# Perceptions and attitudes toward performing risk assessment for periodontal disease: a focus group exploration

**DOI:** 10.1186/s12903-018-0550-2

**Published:** 2018-05-21

**Authors:** Thankam Thyvalikakath, Mei Song, Titus Schleyer

**Affiliations:** 1 Dental Informatics Core, Department of Cariology, Operative Dentistry & Dental Public Health, Indiana University School of Dentistry, Research Scientist, Center for Biomedical Informatics, Regenstrief Institute, Inc, 1050 Wishard Boulevard, R2206, Indianapolis, IN 46202 USA; 2 Microbicide Trials Network, Magee-Womens Research Institute, 204 Craft Avenue, Pittsburgh, PA 15213 USA; 3 Center for Biomedical Informatics, Regenstrief Institute, Inc. Indiana University School of Medicine, 1101 West Tenth Street, Indianapolis, IN 46202 USA

**Keywords:** Risk assessment, Risk assessment tool, Risk factors, Periodontal disease, Electronic health records, Electronic dental records, Dental informatics, Biomedical informatics

## Abstract

**Background:**

Currently, many risk assessment tools are available for clinicians to assess a patient’s periodontal disease risk. Numerous studies demonstrate the potential of these tools to promote preventive management and reduce morbidity due to periodontal disease. Despite these promising results, solo and small group dental practices, where most people receive care, have not adopted risk assessment tools widely, primarily due to lack of studies in these settings. The objective of this study was to explore the knowledge, attitudes, and beliefs of dental providers in these settings toward risk-based care through focus groups.

**Methods:**

We conducted six focus group sessions with 52 dentists and dental hygienists practicing in solo and small group practices in Pittsburgh, PA and New York City (NYC), NY. An experienced moderator and a note-taker conducted the six sessions, each including 8–10 participants and lasting approximately 90 min. All sessions were audio-recorded and transcribed verbatim. Two researchers coded the focus group transcripts. Using a thematic analysis approach, they reviewed the coding results to identify important themes and selected representative excerpts that best described each theme.

**Results:**

Providers strongly believed identifying risk factors could predict periodontal disease and use this information to change their patients’ behavior. A successful risk assessment tool could assist them in educating and changing their patient’s behaviors to adopt a healthy lifestyle, thus enabling them to play a major role in their patients’ overall health. However, to achieve this goal, it is essential to educate all dental providers and not just dentists on performing risk assessment and translating the results into actionable recommendations for patients. According to study participants, the research community has focused more on translating research findings into a risk assessment tool, and less on how clinicians would use these tools during patient encounters and if it affects a patients’ risk or outcome.

**Conclusions:**

Dental practitioners were open to performing risk assessment as routine care and playing a bigger role in their patients’ overall health. Recommendations to overcome major barriers included educating dental providers at all levels, conducting more research about their adoption and use in real-world settings and developing appropriate reimbursement models.

**Electronic supplementary material:**

The online version of this article (10.1186/s12903-018-0550-2) contains supplementary material, which is available to authorized users.

## Background

Periodontal disease is a significant oral health problem induced by dental-plaque bacteria and host-mediated inflammation of the gums. It is the sixth most prevalent chronic condition in the world [1–3] and accounts for approximately 46% of adult periodontitis and 9% of severe periodontal disease in the US [1, 4]. Mounting evidence also suggests a potential association of periodontal disease with systemic diseases such as diabetes, cardiovascular disease, cancer and stroke [1, 5, 6]. Furthermore, poor oral health and tooth loss can negatively affect a person’s self-esteem and overall quality of life [1, 7]. With adults retaining their teeth much longer, oral health remains an important component of their overall health [7]. Unfortunately, many at-risk patients are not identified at the time of routine dental examination. They appear to be in good periodontal health despite the fact they may have underlying risk factors that increase their probability of periodontal disease in the future [8–10].

Until recently, clinicians and researchers considered all people equally susceptible to periodontal disease as most adults suffered from the disease with age [6, 10, 11]. In contrast, it is now a fact that susceptibility to and severity of periodontal disease varies among adults [5]. Many studies have significantly expanded our understanding of the etiology and pathogenesis of periodontal disease. Research has identified many risks and risk-modifying factors [5, 6, 12, 13]. As a result, professional dental associations and dental schools emphasize risk assessment and preventive management for periodontal disease. This approach requires clinicians to perform a thorough patient examination and integrate findings into an accurate and valid assessment of a patient’s current disease status and future disease risk [14].

Currently, many risk assessment tools are available for clinicians to assess a patient’s periodontal disease risk [11, 15–21]. Most tools leverage the information clinicians routinely collect or patients self-report and compute a risk or disease score based on the risk factors. This score is then used to recommend an appropriate treatment plan for the respective patient. A systematic review of these tools indicated their ability to identify subjects with a different probability of periodontitis progression and/or tooth loss in various populations. Other studies demonstrated clinicians’ high agreement with the periodontal diagnosis made by a risk assessment tool [18], their positive attitude towards using such tools [20] and improved patient care outcome and practice productivity [20, 22].

Despite these promising results, solo and small group dental practices, where most people receive care, have not adopted risk assessment tools widely, primarily due to lack of studies focusing on risk assessment in these settings [20]. For instance, limited research has investigated dental practitioners’ perception of risk assessment and incorporation of risk assessment into their daily practice. In addition, while risk assessment may inform patient care, it is not clear how a particular patient may benefit from treatment based on risk assessment [11, 23]. Also, lessons learned in large organizational settings may not be generalizable to small practice settings due to differences in patient characteristics and approaches to providing care [24].

In this study, we explored the views and attitudes of dental practitioners in solo and small-group practices towards a risk-based care approach through focus groups. We also assessed their perceived barriers and facilitators towards using risk assessment tools. Focus groups are an established method to explore the issues affecting novel interventions in clinical practice [25]. The informal and unstructured nature of focus groups helped in elucidating user needs and attitudes and identified significant barriers and opportunities for a risk-based care. Leveraging this knowledge can help better understand the needs of dental practitioners as well as inform the design of future risk assessment tools to improve patient care.

## Methods

### Participant recruitment

We recruited a purposive sample of 52 dental practitioners from Pittsburgh, Pennsylvania (PA), and New York City, New York (NY) and conducted six focus group sessions. In Pittsburgh, we conducted two sessions with dentists and hygienists practicing in urban and suburban areas respectively. In New York City, we conducted one session each with dentists and hygienists. We selected this sample with the intention to explore potential similarities and differences in the research topic between urban and suburban practitioners in academic versus nonacademic settings.

Participants were recruited through local dental study clubs, hygienist associations, University of Pittsburgh School of Dental Medicine part-time faculty list and the Practitioner Engaged in Applied Research & Learning (PEARL) practice-based research network of New York University. These organizations sent an initial email invitation to their members that contained a brief study description, inclusion criteria and an honorarium offer upon complete participation. They also sent a reminder message 2 weeks later. We contacted interested clinicians and confirmed they met the following inclusion criteria: be in active practice (> 30 h/week of clinical activity), graduated from a US-based educational program and be fluent in English.

### Script development

A five-member panel of two dentists, one hygienist and two researchers with qualitative research expertise developed the focus group script. They developed questions geared towards exploring important issues associated with the adoption of a risk-based care for periodontal disease. The questions included dentists’ main focus in the first patient examination; their definition and perception of a risk-based care; current periodontal risk assessment practices; and opinions on the benefits and drawbacks of applying a risk-based approach. We also designed questions to identify major professional, social, cultural, workflow and technical issues that facilitate or hinder the implementation of a risk-based care approach. Additional file 1: Appendix A presents the script used in the focus group sessions. To facilitate the discussion, we used the Previser Risk calculator (PRC) (PreViser Corp., Mount Vernon, WA) [9, 26] as an example to help participants think about risk assessment.

### Focus group sessions

An experienced moderator and a note-taker conducted the six sessions, each including 8–10 participants and lasting approximately 90 min (See Additional file 1: Appendix A). After a short warm-up period, the moderator started the discussion, guiding the questions with the script and a handout of the sample risk assessment tool interface of Previser Risk calculator (PRC) (PreViser Corp., Mount Vernon, WA) [9, 26] when necessary. At the end of each session, participants filled out an exit survey on their practice experience, type of practice, patient population, periodontal charting tools (Additional file 2: Appendix B). The sessions were audio-recorded and transcribed verbatim by professional transcriptionists.

### Coding and data analysis

One informatics researcher (Thankam Paul Thyvalikakath (TPT)), and a qualitative researcher (Mei Song (MS)) coded the focus group transcripts, using the qualitative data analysis software NVivo 10 (QSR International, Australia). They first coded one transcript independently with open coding (the process of selecting and naming categories, and identifying commonalities of the data). They reviewed the codes and discussed any disagreements to reach consensus. Through open coding, they identified a set of higher-level codes to guide subsequent coding. Afterward, they annotated and coded all transcripts using the higher-level codes and sub-codes accordingly. Using a thematic analysis method [27], through constant comparison, the two researchers reviewed the coding results, discussed agreements and resolved discrepancies to identify important themes. They then selected representative excerpts that best described each theme to prepare the study results.

## Results

A total of 27 dentists and 25 hygienists participated in the focus group sessions. The dentist group consisted of 16 male and 11 female participants, and the hygienist group consisted of all female participants. Tables 1 and 2 describe the demographics and patient characteristics of these participants. Our analysis focused on dental practitioners’ perceptions of a risk-based dental care, the ways they performed risk assessment, the benefits of and barriers to performing risk assessment and suggested strategies to overcome barriers. Major themes that emerged from the discussion are described below.

**Table 1:** Characteristics of Focus Group Participants (*n* = 52)

Characteristics	Number	Percent
Gender		
Male	16	(30.8)
Female	36	(69.2)
Position		
Dentist	27	(51.9)
Hygienist	25	(48.1)
Type of Practice^a^		
Solo practice	26	(50.0)
Group practice	14	(26.9)
Community clinic/public health	2	(3.8)
Hospital	3	(5.8)
Other: VA, academic	7	(13.5)
Clinical Experience		
1–10 years	8	(15.4)
11–20 years	10	(19.2)
21–25 years	11	(21.2)
> 25 years	23	(44.2)
Frequency of performing periodontal examination		
Once or more each 6 months	21	(40.4)
At least once a year	24	(46.2)
At least once every 2 years	3	(5.8)
< once every 2 years	1	(1.9)
Never	2	(3.8)
Missing	1	(1.9)
Frequency of referral to periodontist		
0–24%	28	
25–49%	6	
50–74%	7	
75% or more	7	
Worked for a periodontist	3	
Use of Patient Chart^a^		
Paper chart only	15	(28.8)
Computer chart only	8	(15.4)
Both paper and computer chart	20	(38.5)
Unclear	9	(17.3)

^a^Four participants worked at more than one practices, only data applicable to their primary work is counted

**Table 2:** Characteristics of patients treated by the focus group participants (n = 52)

Patient population^a^	Number	Percent
Mainly White patients	27	(51.9)
Mainly African American patients	16	(30.8)
Both White and African American patients	1	(1.9)
Other: Hispanics, Asians	8	(15.4)
Percentage of patients with public insurance		
0–10%	31	(63.46)
More than 10%	4	(7.69)
More than 20%	6	(11.54)
More than 50%	2	(3.86)
Majority of patients are in public programs	9	(17.31)
Number of patients treated with periodontal disease/week		
0–9 patients	32	(61.5)
10–19	11	(21.15)
20–49	8	(15.38)
50 or more	1	(1.92)

^a^Four participants worked at more than one practices, only data applicable to their primary work is counted

### Perceptions of a risk-based care approach

When asked how they defined a risk-based dental care, the majority of providers interpreted it as conducting a certain type of risk assessment for patients. Most commonly, it involved identifying all possible risk factors and assessing their impact on a patient’s oral health. These factors included pre-existing dental problems such as periodontal diseases; medical conditions such as heart diseases, diabetes, high blood pressure; smoking and alcohol drinking; diet habits; and oral hygiene. One hygienist said, “*It’s host resistance. It’s smoking. It’s health. It’s medical condition. It’s psychological condition. They’re all risk factors that you have to either ask or assess with your intuition and kind of put that all together and how individually all those things affect this one person.*”

Providers viewed risk assessment not only on how it affects patients’ ongoing problems but also from the perspective of preventive management. They believed identifying risk factors could predict clinical manifestation of some dental diseases and could use this information to motivate patients for behavior change. One dentist commented, *“From a preventive dentistry standpoint, prevent the progression of the initiation of any kind of dental disease and injury. We do have a lot of patients who are very active and the children come in with a tremendous amount of injuries. We look to see if we can assess what their behaviors are to make sure or at least decrease the possibility of any type of dental disease or pathology, or even injury.”*

Interestingly, a small number of providers (two dentists and two hygienists) perceived risk assessment as evaluating the impact on treatment outcomes. They tried to assess how well a patient would be successful with the proposed treatment given his risk factors. For instance, one dentist said he modified a patient’s treatment based on caries risk assessment: conservative treatment with remineralization in some cases but more aggressive in others. Another dentist described a different scenario, *“If you have somebody that’s 25 years old, and every tooth in their mouth is severely decayed, chances are you being real successful with this patient are somewhat limited, whereas if somebody comes in at 25 and they need one or two small fillings, you can show them why, they can correct their behavior, and they’re gonna be good the rest of their life.”*

For the two hygienists, they seemed to focus on patients’ financial conditions, rather than personal risk factors. For patients who could afford any treatment needed, the risk level for a bad outcome would be low; for those who had no access to care other than free care services, their risk level would be high. Therefore, “*risk-based assessment can mean a lot of different things depending on your setting,”* as one hygienist concluded.

### Current practice of risk-based care

For most study participants, risk assessment was not an intentional and purposeful task they performed for a new patient; instead, they integrated it into the comprehensive examination. Typically, information gathered from the patient brought certain risk factors to the dentist’s attention, such as having diabetes or smoking. During the oral status evaluation, especially the periodontal examination, clinical signs or symptoms triggered the dentist to probe more on potential risk factors. However, dentists did not consider the periodontal evaluation as a form of risk assessment. One dentist noted, “*I don’t call it risk assessment; it’s my periodontal exam. I’m doing the attachment levels, the pocket depths, those types of things. First I do a little mini-screening to see do they need a comprehensive perio exam. If yes, we open that record up and do that.*”

While most dentists used numbers, such as pocket depth and bleeding index, in the periodontal evaluation, they relied mostly on personal knowledge, expertise and practice experience to assess a patient’s oral health and risk factors. With no formal or systematic tools, dentists evaluated risk factors mentally and subjectively. As a dentist summarized, “*Frankly we don’t use tools...all we do is you know, like intuitive finding. You look at the patient, and you get some bells off in your head… I know my knowledge base, I see some signs and symptoms, and then I kind of move on to another thing.*”

A handful of dentists, however, performed risk assessment more systematically, using tools varying from forms to algorithms and software. Two dentists used the American Dental Association’s (ADA) caries risk assessment form and one used caries management by risk assessment form (CAMBRA). One office developed an internal form to assess and assign patients to different risk categories. Another office used a system called Structure Chart, a voice-activated application with a built-in risk assessment function that allows hygienists to record various index, plot periodontal data over time, send updated charting and forms to dentists and deliver a summary report to patients with recommendations. The risk levels, as a hygienist commented, “*were easy to understand and it prompts them to ask questions about their own health, which is good, ‘cause I think it’s more meaningful when it comes from them.*” As a very helpful tool for patient education, this system was well received by hygienists in the office.

### Benefits of performing risk assessment in dental care

To encourage discussion among study participants, we showed them a sample of the risk assessment tool and asked what benefits they perceived it would have on dental care. Thus, the benefits presented here include what they experienced in their practices as well as the perception of a risk assessment tool they may not have used personally.

#### Risk assessment helps dentists practice preventive dentistry

Some dentists felt assessing risk would allow them to identify individual risk factors and categorize patients based on their risk levels for dental diseases. Documentation of this information served as a reminder to dentists during future visits to monitor specific symptoms for preventive care. One dentist in Pittsburgh said it helped him “*set up a checklist so that there is something that I tend to look on occasion; it’s there to remind me to take a good look at that.”* This approach is helpful to prevent dental problems from happening or diagnosing a problem at an early stage so that “*it will be intercepted and treated early, thus earlier treatment, simpler treatment.”*

#### Risk-based care helps dental providers play a bigger role in patients’ health

Providers believed that risk-based dental care could identify not only risk factors for oral diseases but also those affecting a patient’s overall health. As such, assessing risk would help raise the awareness of the oral-systemic connection in the dental community. Subsequently, being mindful of this connection would motivate them to play a bigger role in patients’ health, so that they are “*not just saving teeth and keeping gums healthy, what we do also have an impact on the health of their heart, pancreas, and lungs. It’s really about taking responsibility as a practitioner.”* Taking greater responsibility would also motivate dental practitioners to take a more holistic view of patient health and integrate dental care into medical care. “*It’s a valuable thing to have us as dentists included in the overall health improvement of a patient, and oral health being part of it,”* commented a New York dentist.

#### Risk assessment helps educate patients and dental students

Participants unanimously agreed that a big value of risk-based care is to help them better educate and communicate with patients, especially with the assistance of well-designed and validated risk assessment tools. We provided a sample risk assessment results of the tool that included number scales, charts, graphs and color-coded results. Many participants felt such a tool would be helpful to patients in multiple ways. With the information presented as a summary report, patients can review their results in a concise and easy to understand format. The actionable recommendations are especially valuable to patients who can “*leave with something where they can look at what happened at that visit and know what to do for the future so that they have a plan in their hand.*”

Study participants also considered the tool valuable for educating dental students. It may be debatable to use one numerical score to summarize a patient’s risk level, but from an academic standpoint, some participants believed it could be a useful guideline to learn risk assessment “*especially for the younger dentist going in, first-year or second-year dental students.”* Others shared this view, but considered the tool “*too repetitive*” and less useful for more experienced dentists as they “*may not even need the tool to evaluate (risks) quicker or more concisely.*”

#### Risk-based care helps boost the business of dental practices

In addition to clinical care and education, providers suggested risk-based care has the potential to improve the business side of dentistry. Specifically, performing risk assessment and educating patients would likely improve patients’ satisfaction and acceptance with treatment and increase revenue over time. Being able to categorize patients based on risk level would potentially save hygienists’ time by avoiding unnecessary work for low-risk patients. As one dentist said, “*If you could subcategorize those perio patients, you could save man-hours in time and submitting to insurance forms. These would all be benefits.*” Other providers proposed a less mentioned benefit from a liability standpoint. A few dentists expressed concern that sometimes patients suspected being gouged when receiving more treatment. If dentists “*had some solid numbers documented in the records,*” they would have some proof in hand so the patient “*would have no way out.*”

### Barriers to performing risk assessment in dental care

Providers agreed that performing risk assessment would be useful to improving patient care; however, they identified five barriers that deterred this practice in dental offices. These encompassed various aspects of dental practice, from the nature of performing risk assessment, acceptance by providers, to patients’ reaction and financial considerations.

#### Risk assessment tools lack scientific validation

The most reported barrier concerned the quality of risk assessment tools. Though several tools are available on the market, providers were concerned about the science behind them and the accuracy and validity of the risk assessment. For instance, commenting on the sample tool, one dentist said, “*If there were valid science behind it, it would be a very useful tool. I think one of the issues or problems with this particular risk assessment calculator and other ones is that there isn’t enough medicine behind it.*”

To be considered valid, providers wanted to see well designed studies that evaluated these tools and results published in good scientific journals. More importantly, they would be more convinced of the tool’s usefulness if studies could show performing risk assessment “*is a corollary to the success of treatment outcome*.” For periodontal disease, they wanted to see if treatment based on risk assessment would produce positive results, as a dentist asked, “*for the severe category patients, when we do aggressive periodontal therapy and surgery and adjunctive chemotherapeutic perio treatment, do we get positive or negative results?*”

#### Risk assessment is time-consuming and under-reimbursed

As risk assessment involves identifying multiple factors, a comprehensive assessment could be a challenging task. Gathering and documenting all information would require dentists to spend extra time that many could not afford in a regular 30–40 min appointment. To exacerbate the problem, they felt the format and content of risk assessment tools were not very user-friendly. Many preferred to “*just go through a few slides or a few drop down boxes to get the results,*” so if they had to “*answer two or three pages just to get a quantitative number, it’s not worth it, quite honestly,*” as one dentist said. With extra time spent, providers expected reimbursement for performing a risk assessment. Thus a lack of it posed an important barrier to them. One dentist commented, “*It does sound crass, but time is money…Dentistry is my livelihood, not my hobby. So if I get a questionnaire that’s gonna take me an hour to fill out, and the insurance company reimburses me $15.00?*”

#### Implementing risk assessment programs can be costly

While providers showed interest in risk assessment, they were cautious about adding another technology-based tool in the office when some offices do not even have a computer in every operatory. The cost of purchasing and implementing a tool came to their mind immediately, as one dentist posed these questions, “*How much does this cost me? How much training or man-hours is it gonna take to implement this?”* A hygienist added the potential cost of printing out the summary report generated by a tool. “*We know that taking this would be the best tool, but cost-effectiveness is an issue when you’re handing someone two or three pages of anything, because haven’t we all just really been aggravated by the cost of ink?*”

#### Some dentists are resistant to change their practice routine

A few participants reported that some dentists practiced with a relatively fixed mindset and were reluctant to embrace changes in dentistry, be it using an electronic dental record or practicing risk assessment. They are slow to embrace new technologies and new ways of practice. A dentist offered his observation, “*There are many older people that haven’t jumped on the bandwagon ‘cause of the comfort zone of what they’re doing and how they’re generating their money. They’re making their income, so why change that? Why upset that apple cart?*”

#### Changing patient health behaviors is difficult and challenging

For risk-based care to produce the best results, patients need to accept it. However, providers often encountered patients who do not value preventive care. These patients come to a dentist to fix existing problems, not to prevent future diseases. Dentists found it extremely difficult to motivate them to change oral health habits. One dentist shared a conversation with a young patient who smoked since a very early age, “*When I told a young fellow, ‘You have quite a tobacco pouch here. You really need to stop this. You’re 19 years old.’ I got back, ‘Grandma gave me my first chew at four years old. What’s the matter? You’re telling me my grandma doesn’t know what’s going on?’*” Two hygienists experienced similar frustration when trying to refer people to get the periodontal treatment desperately needed, but to no avail, “*It’s been my feeling all along that the people we recommend to go to a periodontal office never go. We can look back on their chart forever. They’re not going.*”

#### Performing risk assessment results in unintended consequences

In addition to the above barriers, providers identified several unintended consequences of performing risk assessment that further discouraged them. One potential drawback is that doing risk assessment requires entering much information into the computer, which consequently reduces the face time with patients. As a result, the dental provider-patient relationship may suffer. Another unintended result could be the misuse of risk assessment results by insurance companies to refuse coverage for treatment. Providers were concerned that insurance companies would challenge the treatment recommended by a tool that may not fit their rules. The third potential outcome is some patients may be shocked and turned off by a high-risk score generated by a risk assessment tool. Dentists worried that these patients could lose hope and stop trying to improve their oral health altogether.

### Facilitators for performing risk-based care

According to study participants, the primary factor for adopting a risk-based care was to educate all stakeholders involved in patient care. Other facilitators included making risk assessment a standard practice and risk assessment tools universally accepted; providing monetary incentives to providers and having access to well-designed and scientifically validated risk assessment tools. We describe them briefly in this section:

#### Promoting risk assessment to all stakeholders in the dental care

Providers believed that education is the key to the adoption of risk-based care. It is especially important to start the education in dental school so “*students will come out of school wanting to do risk assessment as they learned.*” Then they should teach risk assessment to support staff, such as hygienists and dental assistants. Dentists emphasized the need to teach students not only the meaning of risk assessment but also how to interpret numeric results in a meaningful way. As one dentist suggested, “*A number is just a number. But how do you make it relevant to you as a practitioner? How do you make it relevant to the patient? So there comes education again.*” The third piece of the education is patients. Providers hoped to educate patients more on the oral-systemic connections to better understand their risk profile. With education, they expected patients to *“speak the same language, and hopefully, gets a little bit more motivated (about their health).”*

#### Making risk assessment standard and universally acceptable

Providers believed it is imperative that risk assessment is made a standard of care to enable its wide adoption. They expected the endorsement by dental associations to play an important role. For example, to adopt a periodontal risk assessment tool, a dentist suggested, “*if you got the American Academy of Periodontology on board and endorsed it, the ADA would jump on board. And just like PSR (Periodontal Screening and Recording) scores, it would become universally acceptable.”* Once a standard of care, more people will accept the idea and become an adopter. Providers also proposed the additional value of using universally acceptable risk assessment tools for patient care. For instance, when a patient switches a dental office, the new dentist will understand his risk history and profile instantly if the two dentists use the same tool. This universal approach will ease care coordination between different providers.

#### Providing monetary incentives to dental practitioners

As previously described, a major barrier to performing risk assessment was a lack of reimbursement for dentists. Given the pressing time constraints of the current practice environment, providers, especially dentists, were clear that the ability to generate more revenue would be a big facilitator for risk assessment. One dentist said, “*This may sound callous, but overall, the bulk of dentists who practice dentistry as a business look at the business of dentistry different than the practice of dentistry. We like the new remote control whose 50 buttons does the magic thing, but if it isn’t gonna make me money, I’m not going to be quick to jump on the bandwagon.*”

#### Having access to well-designed, scientifically validated and easy to use risk assessment tools

When all other favorable factors are in place, access to well-designed, clinically based and scientifically validated tools becomes the deciding factor for providers to take up risk assessment. Throughout the discussion, they proposed numerous suggestions for designing an ideal tool. As we will address the details of the requirements in a separate article, we provide a concise summary of their opinions here.

According to them, a risk assessment tool should include multiple features and functions, the most important being that it must consider all relevant factors to calculate the risk score. They suggested adding factors, such as patients’ oral pH, personal life and even emotional status such as “a*re they going through a divorce? Is their stress level higher than normal?”* In addition, the tool should emphasize factors linking dental to medical conditions; track patient changes over time; connect other risk assessment tools, such as caries assessment; and provide personalized recommendations to patients. A valid risk assessment tool needs to assign the right weights to different risk factors to calculate an accurate and personalized risk score. After development, the tool should be tested in well-designed scientific studies. When delivering patient results, they expected the summary report to be concise and tailored to each patient, with better data visualization, clearer, and color-coded scales and written in easy to understand grade-level language. For optimal adoption, this tool also *“needs to be incorporated, not in a stand-alone program, but into a computer program that the office is using*” in the workflow, as one hygienist commented.

## Discussion

This study performed a progressive exploration of general dentists’ and hygienists’ perceptions and attitudes regarding a risk-based care approach for periodontal disease. A major finding of this study is the dental providers’ strong emphasis that a successful risk assessment tool would assist them in educating and changing their patient’s behavior to adopt healthy lifestyle behaviors. They expressed risk assessment played a major role in practicing preventive dentistry and enabling patients to have better outcomes. The study participants also highlighted that performing risk assessment could enable them to play a bigger role in their patients’ overall health. To achieve this goal, it is essential to educate all dental providers and not just dentists on performing risk assessment. It is equally important to teach them to interpret and translate the results into actionable recommendations for patients.

We did not detect significant differences between dentists and hygienists in their perceptions and attitudes toward risk-based care. Both groups viewed risk assessment as identifying all possible risk factors and assessing their impact on a patient’s oral health. They also agreed on the various benefits of and barriers to performing risk assessment. However, sometimes they focused on different aspects of risk assessment. For example, dentists focused on treatment outcome when assessing a patient’s risk level while some hygienists looked more at his/her ability to pay for treatment. On the benefits of risk assessment, dentists focused more on performing preventive dentistry and playing a bigger role in patients’ overall health while hygienists focused on educating patients about oral disease and the connection to medical conditions. It is important to note that these differences mainly stem from the respective clinical roles dentists and hygienists play in the office.

Our study also identified key factors to be addressed to make risk assessment part of routine dental care and to promote preventive management. In this section, we propose directions for future research in this field. We hope these suggestions can leverage practitioners’ positive attitudes and address their concerns to promote preventive management of periodontal disease through risk assessment. It is important to note that medicine also faces the same challenges of adopting risk assessment tools observed in dentistry [28–32]. Since dentistry is yet to adopt a risk-based treatment approach widely, it has the advantage of implementing risk assessment tools that are designed based on dental practitioners’ and patients’ needs.

### Developing standardized and reproducible measures to assess risk for periodontal disease

Currently, dental practitioners mostly rely on their expert knowledge and practice experience to evaluate patients’ risk for periodontal disease. In most cases, they consider this intuitive evaluation sufficient but do admit that acquisition of the skill may require years of experience and learning, especially for new dental school graduates. Thus, it is important for clinicians to assess an individual’s periodontal disease risk and status in objective and standardized ways. Future work should focus on developing standardized and reproducible measures for periodontal risk assessment. Over time, such approaches can provide valuable data to conduct comparative effectiveness research that will lay the foundation for evidence-based dentistry.

### Evaluating risk assessment tools to improve process and patient outcomes

In this study, a major barrier to the adoption of current tools is the lack of scientific evaluation. Admittedly, developing a good decision support tool is a difficult endeavor; however, study participants feel that the research community has overemphasized on translating research findings into a risk assessment tool, not on evaluating if and how it can reduce a patient’s risk. We need more research to validate the risk assessment results in clinical settings to answer critical questions, such as how clinicians interpret the risk scores, how they use the results to make treatment decisions, how they educate patients and promote shared decision-making, and ultimately how risk assessment tools improve treatment processes and patient outcomes.

### Exploring new models of payment to perform risk assessment

Both dentists and hygienists are highly concerned about the current reimbursement model that limits them from performing risk assessment and preventive management. This concern clearly shows that developing scientifically valid and easy to use tools alone are insufficient for clinicians to perform risk assessment. It is critical that the dental profession and researchers explore new models of payment to incentivize providers to conduct risk assessment and transition to a preventive model that will reduce dental diseases and dental care costs.

### Developing innovative and integrative approach to collect patient information

Study participants reported the extra time needed to gather and record information as a notable barrier. We need to develop more innovative approaches to reuse existing data from various sources and improve the user-interface of clinical systems. With the huge amount of data available electronically in the electronic dental record (EDR), electronic health record (EHR), and other sources, how to reuse them more efficiently and effectively remains an important research question. We envision that data collection may use an integrative approach, for instance, by combining patient self-reported general health and lifestyle data collected before visits, dental data collected during patient visits, and medical data automatically imported from other sources before or after visits.

### Designing smart tools to help patients track, monitor and change behaviors

A perfect tool may help providers to predict and prevent oral diseases, but it may not guarantee to change patients’ health behaviors. In addition to incorporating an educational component in the risk assessment results, simple-to-use tools such as smartphone apps could be developed to motivate patients to monitor and improve their oral health behaviors.

### Promoting an integrated care and prevention approach

Dentists in the study embraced the idea of playing a role like primary care physicians. As some medical conditions manifest first in the oral cavity, dental practitioners are in a unique position to identify them earlier than physicians. Moreover, oral diseases share many of the same risk factors with medical conditions such as cardiovascular disease, diabetes, respiratory disease and cancer; thus promoting an integrated care and prevention approach is crucial to improving patient outcomes, population health and reducing per capita costs of healthcare. While it is still debatable if dentists should perform certain screening procedures and claim reimbursement, their openness to a bigger role may play a significant part in raising patients’ awareness of oral-systemic connection and facilitating care coordination for their general health. As indicated in the study, participating in patients’ overall health brings providers more career satisfaction than financial gains.

### Limitations

A major limitation of this study is that we recruited a convenience sample of dentists and hygienists from Pittsburgh and New York City. In the selection process, we purposefully sampled dental practitioners from metro vs. suburban areas to explore potential differences among them on the research questions. However, with Pittsburgh and NYC being densely populated metropolitan areas, the point of views of these providers may not reflect views of providers in other parts of the country, especially those in rural areas or working with specific patient populations. Therefore, the results may not be generalizable to all dental practitioners. To generalize our results, we plan to administer a nation-wide survey of dentists and hygienists based on the themes that emerged from this study. Secondly, participation inequality existed in the focus group sessions. In certain sessions, a few active participants contributed more to the discussion on using risk assessment tools. However, this should not be a serious concern since each participant had a different experience in using these tools. We were more interested in identifying what experience they had in risk assessment and less in who had that experience. Finally, opinions on the research questions may change as the tools become more readily available. It will be helpful to conduct a similar study to compare the results.

## Conclusions

Most dentists and hygienists in the study defined risk-based care as identifying all possible risk factors and assessing the impact on patients’ oral diseases. Dental practitioners were open to performing risk assessment as a routine care practice and playing a bigger role in patients’ overall health. But the extra time needed and lack of reimbursement, the difficulty to change provider and patient behaviors, and the limited availability of scientifically validated tools all posed as roadblocks. To overcome these barriers, dental community should work towards the following: educate providers about risk-based care at all levels; promote risk assessment as standard of care; develop innovative and integrative methods to collect patient data; conduct scientific research to validate risk assessment tools and study their adoption and use in real-world settings; and build simple and easy to use electronic tools to motivate changes in oral health behavior.

## Additional files

Additional file 1: **Appendix A** Final Script Used by the Moderator in Focus Group Sessions. The file contains the script used to conduct focus groups as well as guiding questions. (DOCX 15 kb)

Additional file 2: **Appendix B** Exit Survey Conducted at the End of Focus Group Sessions. The file contains the questions administered at the end of the focus group. (DOCX 14 kb)

## Abbreviations

ADA: American dental association
CAMBRA: Caries management by risk assessment
EDR: Electronic dental record
EHR: Electronic health record
MS: Mei Song
NY: New York
NYC: New York City
PA: Pennsylvania
PEARL: Practitioner engaged in applied Research & Learning
PRC: Previser risk calculator
PSR: Periodontal screening and recording
TPT: Thankam Paul Thyvalikakath
WA: Washington

## References

[1] Eke PI, Wei L, Thornton-Evans GO, Borrell LN, Borgnakke WS, Dye B (2016). Risk indicators for periodontitis in US adults: NHANES 2009 to 2012. J Periodontol.

[2] Eke PI, Dye BA, Wei L, Slade GD, Thornton-Evans GO, Borgnakke WS (2015). Update on prevalence of periodontitis in adults in the United States: NHANES 2009 to 2012. J Periodontol.

[3] CDC (2014). Gum Disease Information [Internet].

[4] Hujoel P, Zina L, Cunha-Cruz J, López R (2013). Specific infections as the etiology of destructive periodontal disease: a systematic review. Eur J Oral Sci.

[5] Bouchard P, Carra MC, Boillot A, Mora F, Rangé H (2017). Risk factors in periodontology: a conceptual framework. J Clin Periodontol.

[6] Genco RJ, Borgnakke WS (2013). Risk factors for periodontal disease. Periodontol 2000.

[7] Buset SL, Walter C, Friedmann A, Weiger R, Borgnakke WS, Zitzmann NU (2016). Are periodontal diseases really silent? A systematic review of their effect on quality of life. J Clin Periodontol.

[8] Kye W, Davidson R, Martin J, Engebretson S (2012). Current status of periodontal risk assessment. J Evid Based Dent Pract.

[9] Page RC, Krall EA, Martin J, Mancl L, Garcia RI (2002). Validity and accuracy of a risk calculator in predicting periodontal disease. J Am Dent Assoc.

[10] Douglass CW (2006). Risk assessment and management of periodontal disease. J Am Dent Assoc.

[11] Lang NP, Suvan JE, Tonetti MS (2015). Risk factor assessment tools for the prevention of periodontitis progression a systematic review. J Clin Periodontol.

[12] Van Dyke TE, Sheilesh D (2005). Risk factors for periodontitis. J Int Acad Periodontol.

[13] AlJehani YA (2014). Risk factors of periodontal disease: review of the literature. Int J Dent.

[14] Tonetti MS, Eickholz P, Loos BG, Papapanou P, van der Velden U, Armitage G (2015). Principles in prevention of periodontal diseases. J Clin Periodontol.

[15] Trombelli L, Farina R, Ferrari S, Pasetti P, Calura G (2009). Comparison between two methods for periodontal risk assessment. Minerva Stomatol.

[16] Lang NP, Tonetti MS (2003). Periodontal risk assessment (PRA) for patients in supportive periodontal therapy (SPT). Oral Health Prev Dent.

[17] Page RC, Martin JA, Loeb CF (2005). The oral health information suite (OHIS): its use in the management of periodontal disease. J Dent Educ.

[18] Mullins JM, Even JB, White JM (2016). Periodontal management by risk assessment: a pragmatic approach. J Evid Based Dent Pract.

[19] Garcia RI, Compton R, Dietrich T (2016). Risk assessment and periodontal prevention in primary care. Periodontol 2000.

[20] Mertz E, Bolarinwa O, Wides C, Gregorich S, Simmons K, Vaderhobli R (2015). Provider attitudes toward the implementation of clinical decision support tools in dental practice. J Evid Based Dent Pract.

[21] Chandra RV (2007). Evaluation of a novel periodontal risk assessment model in patients presenting for dental care. Oral Health Prev Dent.

[22] Busby M, Chapple E, Matthews R, Chapple ILC (2013). Practitioner evaluation of a novel online integrated oral health and risk assessment tool: a practice pilot. Br Dent J.

[23] Asimakopoulou K, Newton JT, Daly B, Kutzer Y, Ide M (2015). The effects of providing periodontal disease risk information on psychological outcomes - a randomized controlled trial. J Clin Periodontol.

[24] Brocklehurst PR, Ashley JR, Tickle M (2011). Patient assessment in general dental practice – risk assessment or clinical monitoring?. BDJ.

[25] Sofaer S (2002). Qualitative research methods. Int J Qual Heal Care.

[26] Martin JA, Page RC, Loeb CF, Levi PA (2010). Tooth loss in 776 treated periodontal patients. J Periodontol.

[27] Braun V, Clarke V (2006). Using thematic analysis in psychology. Qual Res Psychol.

[28] Cresswell K, Majeed A, Bates DW, Sheikh A (2012). Computerised decision support systems for healthcare professionals: an interpretative review. Inform Prim Care.

[29] Sittig DF, Wright A, Osheroff JA, Middleton B, Teich JM, Ash JS (2008). Grand challenges in clinical decision support. J Biomed Inform.

[30] Légaré F, Ratté S, Gravel K, Graham ID (2008). Barriers and facilitators to implementing shared decision-making in clinical practice: update of a systematic review of health professionals’ perceptions. Patient Educ Couns.

[31] Carter-Harris L, Gould MK (2017). Multilevel barriers to the successful implementation of lung Cancer screening: why does it have to be so hard?. Ann Am Thorac Soc.

[32] Bates DW, Kuperman GJ, Wang S, Gandhi T, Kittler A, Volk L (2003). Ten commandments for effective clinical decision support: making the practice of evidence-based medicine a reality. J Am Med Inform Assoc.

