# Supplementary material for: Perceptions and attitudes toward performing risk assessment for periodontal disease: a focus group exploration
Source: BMC Oral Health. 2018 May 21;18:90. doi: 10.1186/s12903-018-0550-2 (PMC5963023; doi:10.1186/s12903-018-0550-2)
Supplement: Supplementary file 1 — Appendix A Final Script Used by the Moderator in Focus Group Sessions. The file contains the script used to conduct focus groups as well as guiding questions. (DOCX 15 kb) [file 12903_2018_550_MOESM1_ESM.docx]

**Appendix A: Final Script Used by the Moderator in Focus Group Sessions**

Introduction:

Thank you for coming to this focus group discussion. My name is __________, and I will be your facilitator for today's session. You are taking part in a research study to explore your attitudes and opinions about using risk assessment tools in practice. This discussion will be approximately 11/2 hours long, and it will be audio recorded so that we can recall exactly what was said.

Since we are interested in hearing your experiences and opinions, you are the experts in this discussion. There is no right or wrong answer to any of the questions that I will be asking today. Different points of view are very welcome, so please chime in if you take a different stance on an issue.

To keep this conservation running smoothly, I ask that you speak one at a time, especially since our audio record is very sensitive and often picks up background noise. If you do not understand the questions I am asking at any time, please feel free to ask me for clarification. If I have not heard from someone in a while, I may call on you because your feedback is very important to us and we want to make sure we get everyone's thoughts and opinions. Please respect other people's privacy by not discussing the comments you hear today with anyone else.

I would like to introduce ________; she will be taking notes during the discussion but will not participate in the conversation. Remember that your participation is completely voluntary and you may withdraw from this project at any time. I will not take offense if you do not feel comfortable talking about something or if you leave early. For your generosity with your time and to assist you with travel expenses, you will be given a $XX gift card at the end of the session. This focus group will end promptly at X:00. Are there any questions before I begin?

Questions

1. Let's go around the table and introduce yourselves by the first name only, and tell us why you chose dentistry as a profession, where you went to school, how long you have been in practice. Tell us about your practice.
2. What is your main focus when examining a first-time patient?
3. What is most important to you when treating a patient? What other things do you focus when you are examining any patient?
4. For starters, how would you define a risk-based care approach in dentistry?
5. What are your thoughts on using a risk-based care for periodontal disease?

Do you use or have you thought about using a risk-based approach or risk assessment in your own practice?

- - (If so) – How would you or do you do it?
  - How would you or do you fit this into your workflow?
  - In which situations?
  - Any specific tools?
  - Are you aware of any specific tools on the market now for risk assessment? If so, what do you think of them? (show tools here) What do you think of this specific risk assessment program from what you can tell from these screenshots?
  - How do you think patients react or will react to using risk-based approach?
  - Do you use any electronic patient record? What are your thoughts on these?
  - What are your thoughts on incorporating a risk assessment tool into an electronic patient records system?
- Who else in your office does or could do risk-assessment? (examples – dental hygienists, dental assistants?)

1. What are the benefits you see in using a risk-based approach in dentistry in general and in your practice specifically?
2. What are the drawbacks to using a risk-based approach in dentistry in general and in your practice specifically?
3. What issues, problems or barriers do you see in using a risk assessment approach?
4. What are some suggestions on how these barriers or drawbacks could be reduced or resolved?
5. What might encourage you to use risk assessment in your own practice?

Imagine an ideal world where you are already using risk assessment - picture yourself using risk assessment, and describe what the situation and the tools would be like.
