# Supplementary material for: Perceptions and attitudes toward performing risk assessment for periodontal disease: a focus group exploration
Source: BMC Oral Health. 2018 May 21;18:90. doi: 10.1186/s12903-018-0550-2 (PMC5963023; doi:10.1186/s12903-018-0550-2)
Supplement: Supplementary file 2 — Appendix B Exit Survey Conducted at the End of Focus Group Sessions. The file contains the questions administered at the end of the focus group. (DOCX 14 kb) [file 12903_2018_550_MOESM2_ESM.docx]

**Appendix B: Exit Survey Conducted at the End of Focus Group Sessions**

1. Are you a (please check your current position)

- Dental practitioner
- Dental Hygienist

1. How many years of clinical experience do you have? _______
2. Do you work in (please select one option):

- Solo practice
- Group practice
- Community clinic/public health
- Hospital
- Other (please specify): _________________

1. When did you graduate from dental or dental hygiene school?
2. Name of school__________________

Year of most recent graduation ______________________

Name of most recent dental or dental hygiene school _____________________

1. Your gender

- Male
- Female

1. How best would you describe the demographics of your patients?

- Mainly White patients
- Mainly African American patients
- Both White patients and African American patients
- Other (please specify): _________

1. What proportion of your patients is in a public insurance program such as Medicaid?

- 0% to 10%
- More than 10%
- More than 20%
- More than 50%
- Majority (more than 50%) of patients are in public programs

1. How frequently do you manage patients with evidence of periodontal disease? For example, you and your hygienists perform scaling and root planing on ______ # of patients per week.

- 0-9 patients per week
- 10-19 patients per week
- 20-49 patients per week
- 50 or more patients per week

1. How frequently do you perform comprehensive periodontal examinations (probing of six sites of every erupted tooth) on your patients?

- Every six months or more
- Less often than every six months, but at least once each year
- Less often than once each year, but at least every two years
- Less often than once every two years
- I do not perform these examinations routinely on my patients

1. When you perform comprehensive periodontal examinations as in the previous question, do you record bleeding sites for each erupted tooth**?**

- Yes
- No
- I do not perform these examinations routinely on my patients

1. What percentage of your patients who have the periodontal disease do you refer to periodontal specialists?

- 0-24%
- 25-49%
- 50-74%
- 75% or more

1. Do you use a paper chart or computer chart to manage clinical (as opposed to administrative) patient data?

- Paper chart only
- Computer chart only
- I use both a paper chart and computer

1. If you use a computer to chart clinical patient data, what manufacturer/brand do you use?

- Dentrix
- SoftDent EagleSoft
- EagleDental
- Practice Works
- Other, please record type here:

1. Would you be willing to try a computer-based risk assessment tool to assess the risk of patients for periodontal disease?

- yes
- no
